# Could a Personalized Strategy Using Accelerated Partial Breast Irradiation be an Advantage for Elderly Patients? A Systematic Review of the Literature and Multidisciplinary Opinion

**DOI:** 10.1155/2020/3928976

**Published:** 2020-02-28

**Authors:** Luca Tagliaferri, Valentina Lancellotta, Giuseppe Colloca, Fabio Marazzi, Valeria Masiello, Giorgia Garganese, György Kovács, Vincenzo Valentini, Maria Antonietta Gambacorta

**Affiliations:** ^1^UOC Radioterapia Oncologica, Dipartimento di Diagnostica per Immagini, Radioterapia Oncologica ed Ematologia, Fondazione Policlinico Universitario A. Gemelli IRCCS, Rome, Italy; ^2^Dipartimento Scienze della Salute della Donna, del Bambino e di Sanità Pubblica, Fondazione Policlinico Universitario A. Gemelli IRCCS, Rome, Italy; ^3^Mater Olbia Hospital, Gynecology and Breast Care Center, Olbia, Italy; ^4^Gemelli Interacts, Università Cattolica del Sacro Cuore, Rome, Italy; ^5^Università Cattolica del Sacro Cuore, Istituto di Radiologia, Rome, Italy

## Abstract

*Introduction*. Elderly patients are underrepresented from a majority of clinical trials and the choice of the best treatment becomes a challenge. The optimal treatment should be personalized and based on a multidisciplinary approach that includes radiation oncologists, surgeons, geriatricians, medical oncologists, social workers, and support services. The global evaluation of the patients and the creation of nomograms may facilitate the definition of long-term treatment benefits minimizing the use of unnecessary therapy. *Material and Method*. A systematic research using PubMed, Scopus, and Cochrane library was performed to identify full articles analyzing the efficacy of APBI in elderly patients with breast cancer. ClinicalTrials.gov was searched for ongoing or recently completed trials, and PROSPERO was searched for ongoing or recently completed systematic reviews.

**Results:**

Seven papers fulfilled the eligibility criteria. The number of evaluated patients was 405 and the median age was 77.7 years. The disease-free survival (DFS) range was 96.1%–100%, the grade 3-4 toxicity range was 0%–6.6%, the cancer-specific survival (CSS) range was 97.9%–100%, and the overall survival (OS) range was 87%–100%. All studies reported excellent/good cosmetic results in a range of 74% to 99%.

**Conclusion:**

Accelerated partial breast irradiation (APBI) results in a safe and effective substitute for the adjuvant external beam radiotherapy in selected elderly early-stage breast cancer patients. Based on the relatively low toxicity, APBI should be advised in selected patients with life expectancies larger than 5–10 years.

## 1. Introduction

Breast cancer is the most common cancer in women, and the risk to develop breast cancer increases with age. Indeed 21% of all cases and 13% of breast cancer mortality occur in patients aged ≥70 years old [1]. Despite this data, elderly patients are underrepresented from a majority of clinical trials and the choice of the best treatment becomes a challenge. A great need remains for studies providing evidence levels to guide the treatment of elderly patients, which is often not guideline adherent. Patients aged 70 years and over, who are in good health condition, have a median life expectancy of 15.5 years and half of them will live much longer. Treatment decisions should not be based on age alone but need to ensure that older patients get the best quality of care [2, 3]. There is growing awareness that functional age is a more accurate indicator of cancer treatment compliance because it differs between patients with the same chronologic age [4, 5]. Furthermore, consensus guidelines and position statements recommend the use of the geriatric assessment in elderly patients with cancer [6, 7] in order to avoid worsening of global quality of life.

The optimal treatment should be personalized [8–11] and based on a multidisciplinary approach that includes radiation oncologists, surgeons, geriatricians, medical oncologists, social workers, and support services. In this way, we can obtain an informed discussion of the estimated benefits and risks of cancer treatment. The global evaluation of the patients and the creation of nomograms [12, 13] may facilitate the definition of long-term treatment benefits minimizing the use of unnecessary therapy.

Several randomized trials [14–17] have shown the safety of omitting radiotherapy, however, with little impact on clinical practice [18–20], because there are subgroups of fit older patients where radiotherapy cannot be systematically omitted [21–23]. The impact of local relapse on quality of life should be considered when radiotherapy is intended to omit [24, 25]. To overcome this problem and to prevent undertreatment, accelerated partial breast irradiation (APBI) can be considered an alternative to conventional external beam radiotherapy or exclusive hormonal therapy because it improves convenience for women with low-risk tumors [26–35]. Moreover, the side effects of hormonal therapy can modify the quality of life and patients' reported outcomes during follow-up without a real benefit on overall survival [26–35].

The present systematic review was performed to assess the effectiveness and outcomes of APBI in the adjuvant treatment of elderly patients with breast cancer.

## 2. Materials and Methods

A systematic research using PubMed, Scopus, and Cochrane library was performed to identify full articles analyzing the efficacy of APBI in elderly patients with breast cancer. ClinicalTrials.gov was searched for ongoing or recently completed trials, and PROSPERO was searched for ongoing or recently completed systematic reviews. The studies were identified through the following medical subject headings (MeSH) and keywords including ‘‘breast cancer”, ‘‘brachytherapy”, ‘‘elderly”, and “palliation”. The search was restricted to the English language. The Medline search strategy was (“Brachytherapy” [Mesh] OR ‘‘Brachytherapy” [All Fields]) AND ('‘Breast Neoplasms” [Mesh] OR ‘‘Breast neoplasms” [All Fields] AND “Aged” [Mesh] OR “Aged” [All Fields]). To avoid missing relevant studies, we chose this strategy with high sensitivity but low specificity.

We analyzed only clinical full-text studies of elderly breast cancer patients treated with APBI alone. Conference papers, surveys, letters, editorials, book chapters, and reviews were excluded. Time restriction (1990–2018) as concerns the years of the publication was considered.

Two independent radiation oncologists expert in radiotherapy for breast cancer (VL expert in interventional radiotherapy and VM expert in external beam radiotherapy) screened citations in titles and abstracts to identify appropriate papers. Eligible citations were retrieved for full-text review. Uncertainties about their inclusion in the review were controlled by an expert multidisciplinary team composed by a radiation oncologist expert in interventional radiotherapy of another institution (GK), a surgeon (GG), a medical and radiation oncologist (FM), and a geriatric (GC). Finally, an expert committee (VV, MAG, and LT) performed an independent check and the definitive approval of the review.

The primary outcome was the disease-free survival after APBI during follow-up. Secondary outcomes included specific cancer survival, overall survival, and adverse event rates.

A summary table (Table 1) was created including mono/multicentric study, sample size, median age, disease-free survival (DFS), toxicity, cancer-specific survival (CSS), and overall survival (OS).

## 3. Results

**Figure 1:**
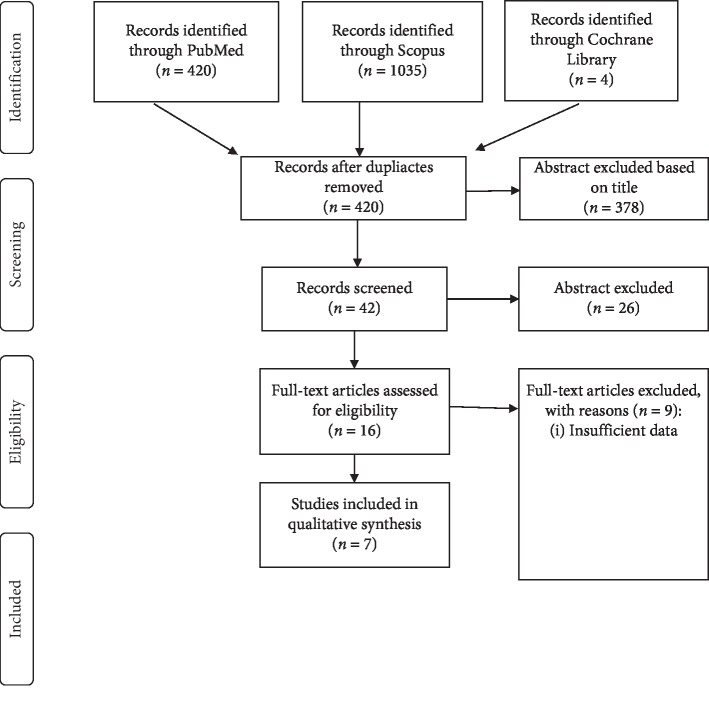
PRISMA flowchart for the outcome and late adverse effects.

The literature search resulted in 420 articles (Figure 1). After the screening of the titles, abstracts, and language of these references, 378 studies were excluded, and 16 full-text articles were selected. Of these, 7 papers fulfilled the eligibility criteria.

Only one study is randomized [36], two studies are phase II [37, 38], and 4 studies are retrospective investigations [39–42]. Following the defined selection criteria, only data from the APBI treatments in elderly patients were extracted and considered for the analysis. The number of evaluated patients was 405 and the median age was 77.7 years. The DFS range was 96.1%–100%, the grade 3-4 toxicity range was 0%–6.6%, the CSS range was 97.9%–100%, and the OS range was 87%–100%.

Table 1 lists the characteristics of the included studies.

All studies reported excellent/good cosmetic results in a range of 74% to 99%.

## 4. Discussion

Elderly breast cancer represents one of the main public health issues, which will become more critical with the increasing life expectancy. Patients aged 70 years and over who are in good health condition have a median life expectancy of 15.5 years and half of them will live much longer [43, 44].

Optimal treatment decisions should not be based on chronological age alone, but need to ensure that elderly patients get the best possible quality of care. The presence of other characteristics (concurrent comorbid illnesses) that represent potential causes of mortality must also be considered to identify those women who are unlikely to die of breast cancer and for whom the omission of adjuvant treatment may be the best option. A geriatric assessment should be mandatory because it provides specific and overall information about the health status, focusing on somatic, functional, and psychosocial domains, which is necessary to provide a multidisciplinary treatment plan [45]. Unfortunately, only a limited number of studies have focused on the role of geriatric assessment in treatment decisions for older early breast cancer patients [46]. It is well known that elderly women are undertreated with breast-conserving surgery (BCS) in favor of mastectomy [47, 48]. There are many reasons for this, such as logistical concerns related to radiotherapy, the possibility of comorbidity, functional impairment, frailty, ideas about body-image, among others.

Furthermore, relatively few elderly patients are accrued in clinical trials. Barriers to the accrual of elders include “physician bias” based on the fear that the patient will not tolerate or will not benefit from the treatment and “patient and family members bias” based on the belief that treatment is not worthwhile or is too toxic. The treatment of elderly breast cancer patients often does not comply with guidelines and older women may receive less of adjuvant radiotherapy following BCS and variably more hormonal therapy. For this reason, for elderly patients with early breast cancer, the choice of the appropriate adjuvant treatment remains challenging.

Multiple trials over the years have investigated the role of RT and TAM in low-risk breast cancer patients in terms of local recurrence (LR), metastases disease-free survival (DMFS), and OS [49–53] and the possibility of avoiding RT [14–17]. Sole adjuvant endocrine or radiotherapy seems to be equivalent to all important oncological endpoints. Endocrine therapy is frequently associated with fatigue symptoms and possible severe side effects like thromboembolic events, endometrial cancer related to tamoxifen, as well as osteoporosis, cardiovascular disease, and arthralgia related to aromatase inhibitors. In contrast to radiotherapy at least 5 years lasting endocrine therapy, the typical side effects of a three-week hypofractionated adjuvant radiotherapy course, like low-grade erythemas and minor edemas, appear relatively moderated and are of short duration [54]. Moreover, it is important to keep in mind that personalized treatment decisions should be based on the patient's baseline risk of recurrence. Indeed, there is strong evidence that the addition of radiotherapy reduces the risk of breast and axillary recurrence and this effect is maintained at 10 years, while radiotherapy does not significantly affect distant recurrence or overall survival rates in patients over 70 years old [14–17].

Moreover, it is important to consider the adherence of patients to endocrine therapy: given the associated adverse effects including hot flashes, thrombotic events, bone loss, and joint pain/stiffness, several studies reported a dropdown of treatment adherence up to the rate of 67% in the first year of treatment, with a further reduction up to 30% in the fifth year [55, 56]. In the longer term, the potential effect of a local relapse on quality of life and the psychological state in older patients should not be underestimated.

An important issue to consider is that not all patients 70 or older are the same. A healthy 70-year-old woman has a high chance of living for more than 10 years, risking a one-in-10 rate of local recurrence if radiotherapy is omitted and a one-in-50 rate if radiotherapy is given. Conversely, in patients with significant comorbidities, the benefit of endocrine therapy can be questioned: the survival benefit of systemic treatment in patients with low-risk tumors is seen after 5 years, whereas the benefit of radiotherapy in reducing local recurrence is considerable in the first 5 years with a survival benefit at 15 years. In this subgroup of patients, perhaps endocrine therapy and not radiotherapy can be omitted.

The omission of radiotherapy may be proposed in low-risk breast cancer patients with limited life expectancy below 5 years. As most patients will present with substantially longer life expectancies, individual counseling about the risks and benefits of radiotherapy, based on clinical and biological features, is strongly recommended. Thus, there is no subgroup of fit older patients in whom post-BCS RT can be systematically omitted.

Since resistance to RT omission persists even in selected cases due to the risk of local recurrence and the availability of alternative forms of RT, APBI may consider a valuable compromise between EBRT and exclusive endocrine therapy.

Particularly in elderly patients, APBI presents many advantages like the possibility of delivering higher doses in the area of the tumor bed and in the same time reducing the dose to the normal breast tissue and adjacent organs at risk [57]. Additionally, a shorter treatment time might improve the convenience and quality of life of the patients, possibly reducing the physical and psychological stress related to radiotherapy [58–60]. These all increase their adherence and reduce the likelihood of inappropriate mastectomy [61–63]. Finally, APBI may reduce the total costs of treatment depending on the used modality [64]. There are many available techniques to perform APBI including intraoperative radiotherapy (IORT), three-dimensional or intensity-modulated EBRT involving stereotactic capabilities, or interventional radiotherapy (IRT). Careful patient selection is an important element to define which patients are suitable for APBI. Four published consensus statement criteria can help the radiation oncologist in this choice (ASTRO: American Society for Radiation Oncology; GEC-ESTRO: Groupe Europeén de Curiethérapie-European Society for Radiotherapy and Oncology; ABS: American Brachytherapy Society; ASBS: American Society of Breast Surgeons) [26–29]. Four phase III randomized trials on APBI have been published up to date, but none of these was specifically designed for older women [30, 31, 33, 34], and no comparison can be made with other trials in which the omission of radiation therapy has been investigated [15].

The 7 studies reported in this review showed excellent rates of DFS (range 96%–100%), CSS (97.9%–100%), and OS (87%–100%) with acceptable G3 toxicities (range 0%–6.6%). Moreover, all studies reported an excellent/good cosmetic result range 74%–99%.

Personalized assessments of the risk benefits are essential when considering older patients for adjuvant treatment after breast-conserving surgery. The use of large databases and nomogram could help for improved analysis of the outcomes in these populations [65–69].

## 5. Conclusions

APBI results in a safe and effective substitute for the adjuvant EBRT in selected elderly early-stage breast cancer patients. It is more convenient for high-volume radiation centers with long waiting lists and for patients who live far away from RT centers. Radiotherapy departments have to be aware of this issue, to provide the best therapeutic option combining optimal local control with good quality of life in a cost-effective way. The actual choice of APBI techniques will be influenced by many factors whereby the strongest arguments are local experience and hospital budget size.

However, one of the most important considerations is to select the most appropriate patient population for this treatment strategy and this should be performed in experienced and trained hands.

In aged or frail patients, a comprehensive assessment of the overall health status is recommended when weighing the expected absolute benefits of cancer treatment against tumor biology, potential toxicities, physiological age, patient preference, quality of life, and remaining life expectancy.

Based on the relatively low toxicity, APBI should be advised in selected patients with life expectancies larger than 5–10 years.

## Figures and Tables

**Table 1:** Characteristics of the included studies.

Author	Period	Study	Sample size, *n*	APBI	Median age, years	DFS	Toxicity (G3-G4)	CSS	OS
Cozzi et al. [39]	2006–17	Retrospective	86	HDR-IRT	82 (44–92)	Recurrent at 3 years: 96% Primary at 3 years: 97.8%	5.6%	100% at 3 years	Recurrent at 3 years: 87% Primary at 3 years: 89%
Genebes et al. [40]	2005–16	Retrospective	70	HDR-IRT	80.7 (62–93.1)	97.6% at 5 years	0%	97.9% at 5 years	93.2% at 5 years
Hannoun-Lévi et al. [37]	2012–14	Phase II	26	HDR-IRT	77 (69–89)	100% at 3 years	0%	100% at 3 years	95.2% at 3 years
Hannoun-Lévi et al. [38]	2004–08	Phase II	40	HDR-IRT	74 (70–87)		2%	100% at 3 years	100% at 3 years
Kinj et al. [41]	2012–15	Retrospective	45	HDR-IRT	77.7 (65–92)	100%,	6.6%	100%, at 3 years	93.1% at 3 years
Meattini et al. [36]	2005–13	Randomized phase 3	58 EBRT 59 APBI	IMRT	EBRT 74.1 (70.0–83.2) APBI 74.4 (70.1–85.3)	EBRT 96.1% at 5 years APBI 98.1% at 5 years	EBRT 5.1% APBI 1.7%	100% at 5 years	
Sumodhee et al. [42]	2005–16	Retrospective	79	HDR-IRT	77 (66–89)	97.4% at 10 years	0%	98.1%	

APBI: accelerated partial breast irradiation; HDR-IRT: high dose rate interventional radiotherapy; IMRT: intensity-modulated radiotherapy; EBRT: external beam radiotherapy; DFS: disease-free survival; CSS: cancer specific survival; OS: overall survival.

## References

[1] Siegel R., Naishadham D., Jemal A. (2012). Cancer statistics, 2012. *CA: A Cancer Journal for Clinicians*.

[2] Mohile S. G., Magnuson A. (2013). Comprehensive geriatric assessment in oncology. *Cancer and Aging*.

[3] Fried L. P., Tangen C. M., Walston J. (2001). Frailty in older adults: evidence for a phenotype. *The Journals of Gerontology Series A: Biological Sciences and Medical Sciences*.

[4] Hurria A., Lachs M. S., Cohen H. J., Muss H. B., Kornblith A. B. (2006). Geriatric assessment for oncologists: rationale and future directions. *Critical Reviews in Oncology/Hematology*.

[5] Pallis A. G., Fortpied C., Wedding U. (2010). EORTC elderly task force position paper: approach to the older cancer patient. *European Journal of Cancer*.

[6] Decoster L., Van Puyvelde K., Mohile S. (2015). Screening tools for multidimensional health problems warranting a geriatric assessment in older cancer patients: an update on SIOG recommendations. *Annals of Oncology*.

[7] Mohile S. G., Dale W., Somerfield M. R. (2018). Practical assessment and Management of vulnerabilities in older patients receiving chemotherapy: ASCO guideline for geriatric oncology. *Journal of Clinical Oncology*.

[8] Lancellotta V., Kovács G., Tagliaferri L. (2019). The role of personalized Interventional Radiotherapy (brachytherapy) in the management of older patients with non-melanoma skin cancer. *Journal of Geriatric Oncology*.

[9] Lancellotta V., Kovács G., Tagliaferri L. (2018). Age is not a limiting factor in interventional radiotherapy (brachytherapy) for patients with localized cancer. *BioMed Research International*.

[10] Kovács G., Tagliaferri L., Valentini V. (2017). Is an interventional oncology center an advantage in the service of cancer patients or in the education? the gemelli hospital and Interacts experience. *Journal of Contemporary Brachytherapy*.

[11] Aristei C., Lancellotta V., Piergentini M. (2019). Individualized 3D-printed templates for high-dose-rate interstitial multicathether brachytherapy in patients with breast cancer. *Brachytherapy*.

[12] Tagliaferri L., Pagliara M. M., Masciocchi C. (2017). Nomogram for predicting radiation maculopathy in patients treated with Ruthenium-106 plaque brachytherapy for uveal melanoma. *Journal of Contemporary Brachytherapy*.

[13] Albert J. M., Liu D. D., Shen Y. (2012). Nomogram to predict the benefit of radiation for older patients with breast cancer treated with conservative surgery. *Journal of Clinical Oncology*.

[14] Kunkler I. H., Williams L. J., Jack W. J. L., Cameron D. A., Dixon J. M. (2015). Breast-conserving surgery with or without irradiation in women aged 65 years or older with early breast cancer (PRIME II): a randomised controlled trial. *The Lancet Oncology*.

[15] Hughes K. S., Schnaper L. A., Berry D. (2004). Lumpectomy plus tamoxifen with or without irradiation in women 70 years of age or older with early breast cancer. *New England Journal of Medicine*.

[16] Fyles A. W., McCready D. R., Manchul L. A. (2004). Tamoxifen with or without breast irradiation in women 50 years of age or older with early breast cancer. *New England Journal of Medicine*.

[17] Fisher B., Bryant J., Dignam J. J. (2002). Tamoxifen, radiation therapy, or both for prevention of ipsilateral breast tumor recurrence after lumpectomy in women with invasive breast cancers of one centimeter or less. *Journal of Clinical Oncology*.

[18] Tinterri C., Gatzemeier W., Zanini V. (2009). Conservative surgery with and without radiotherapy in elderly patients with early-stage breast cancer: a prospective randomised multicentre trial. *The Breast*.

[19] Pötter R., Gnant M., Kwasny W. (2007). Lumpectomy plus tamoxifen or anastrozole with or without whole breast irradiation in women with favorable early breast cancer. *International Journal of Radiation Oncology∗Biology∗Physics*.

[20] Soulos P. R., Yu J. B., Roberts K. B. (2012). Assessing the impact of a cooperative group trial on breast cancer care in the medicare population. *Journal of Clinical Oncology*.

[21] Bellon J. R., Harris E. E. R., Arthur D. W. (2011). ACR appropriateness criteria conservative surgery and radiation-stage I and II breast carcinoma. *The Breast Journal*.

[22] Hurria A., Browner I. S., Cohen H. J. (2012). Senior adult oncology. *Journal of the National Comprehensive Cancer Network*.

[23] Biganzoli L., Wildiers H., Oakman C. (2012). Management of elderly patients with breast cancer: updated recommendations of the international society of geriatric oncology (SIOG) and European society of breast cancer specialists (EUSOMA). *Lancet Oncol*.

[24] Perrucci E., Lancellotta V., Bini V. (2015). Quality of life and cosmesis after breast cancer: whole breast radiotherapy vs partial breast high-dose-rate brachytherapy. *Tumori Journal*.

[25] Schäfer R., Strnad V., Polgár C. (2018). Quality-of-life results for accelerated partial breast irradiation with interstitial brachytherapy versus whole-breast irradiation in early breast cancer after breast-conserving surgery (GEC-ESTRO): 5-year results of a randomised, phase 3 trial. *The Lancet Oncology*.

[26] Husain Z. A., Mahmood U., Hanlon A. (2011). Accelerated partial breast irradiation via brachytherapy: a patterns-of-care analysis with ASTRO consensus statement groupings. *Brachytherapy*.

[27] Polgár C., Limbergen E. V., Pötter R. (2010). Patient selection for accelerated partial-breast irradiation (APBI) after breast-conserving surgery: recommendations of the Groupe Européen de Curiethérapie-European Society for Therapeutic Radiology and Oncology (GEC-ESTRO) breast cancer working group based on clinical evidence (2009). *Radiotherapy and Oncology*.

[28] Shah C., Vicini F., Wazer D. E., Arthur D., Patel R. R. (2013). The American Brachytherapy Society consensus statement for accelerated partial breast irradiation. *Brachytherapy*.

[29] Correa C., Harris E. E., Leonardi M. C. (2017). Accelerated partial breast irradiation: executive summary for the update of an ASTRO evidence-based consensus statement. *Practical Radiation Oncology*.

[30] Strnad V., Ott O. J., Hildebrandt G. (2016). 5-year results of accelerated partial breast irradiation using sole interstitial multicatheter brachytherapy versus whole-breast irradiation with boost after breast-conserving surgery for low-risk invasive and in-situ carcinoma of the female breast: a randomised, phase 3, non-inferiority trial. *The Lancet*.

[31] Livi L., Buonamici F. B., Simontacchi G. (2010). Accelerated partial breast irradiation with IMRT: new technical approach and interim analysis of acute toxicity in a phase III randomized clinical trial. *International Journal of Radiation Oncology∗Biology∗Physics*.

[32] Olivotto I. A., Whelan T. J., Parpia S. (2013). Interim cosmetic and toxicity results from RAPID: a randomized trial of accelerated partial breast irradiation using three-dimensional conformal external beam radiation therapy. *Journal of Clinical Oncology*.

[33] Vaidya J. S., Joseph D. J., Tobias J. S. (2010). Targeted intraoperative radiotherapy versus whole breast radiotherapy for breast cancer (TARGIT-A trial): an international, prospective, randomised, non-inferiority phase 3 trial. *The Lancet*.

[34] Veronesi U., Orecchia R., Maisonneuve P. (2013). Intraoperative radiotherapy versus external radiotherapy for early breast cancer (ELIOT): a randomised controlled equivalence trial. *The Lancet Oncology*.

[35] Aristei C., Maranzano E., Lancellotta V. (2017). Partial breast irradiation with interstitial multi-catheter high-dose-rate brachytherapy. Long-term results of a phase II prospective study. *Radiotherapy and Oncology*.

[36] Meattini I., Saieva C., Marrazzo L. (2015). Accelerated partial breast irradiation using intensity-modulated radiotherapy technique compared to whole breast irradiation for patients aged 70 years or older: subgroup analysis from a randomized phase 3 trial. *Breast Cancer Research and Treatment*.

[37] Hannoun-Lévi J.-M., Cham Kee D. L., Gal J. (2018). Accelerated partial breast irradiation for suitable elderly women using a single fraction of multicatheter interstitial high-dose-rate brachytherapy: early results of the Single-Fraction Elderly Breast Irradiation (SiFEBI) Phase I/II trial. *Brachytherapy*.

[38] Hannoun-Levi J.-M., Gourgou-Bourgade S., Belkacemi Y. (2013). GERICO-03 phase II trial of accelerated and partial breast irradiation in elderly women: feasibility, reproducibility, and impact on functional status. *Brachytherapy*.

[39] Cozzi S., Laplana M., Najjari D. (2018). Advantages of intraoperative implant for interstitial brachytherapy for accelerated partial breast irradiation either frail patients with early-stage disease or in locally recurrent breast cancer. *Journal of Contemporary Brachytherapy*.

[40] Genebes C., Chand M.-E., Gal J. (2014). Accelerated partial breast irradiation in the elderly: 5-year results of high-dose rate multi-catheter brachytherapy. *Radiation Oncology*.

[41] Kinj R., Chand M. E., Jocelyn G. (2018). Single fraction of accelerated partial breast irradiation in the elderly: early clinical outcome. *Radiation Oncology*.

[42] Sumodhee S., Levy J., Chamorey E. (2017). Accelerated partial breast irradiation for elderly women with early breast cancer: a compromise between whole breast irradiation and omission of radiotherapy. *Brachytherapy*.

[43] Pawlik T., Buchholz T. A., Kuerer H. M. (2004). The biologic rationale for and emerging role of accelerated partial breast irradiation for breast cancer. *Journal of the American College of Surgeons*.

[44] Hershman D. L., Buono D., McBride R. B. (2008). Surgeon characteristics and receipt of adjuvant radiotherapy in women with breast cancer. *JNCI: Journal of the National Cancer Institute*.

[45] Extermann M., Aapro M., Bernabei R. (2005). Use of comprehensive geriatric assessment in older cancer patients:. *Critical Reviews in Oncology/Hematology*.

[46] Parks R. M., Lakshmanan R., Winterbottom L., Al Morgan D., Cox K., Cheung K. L. (2012). Comprehensive geriatric assessment for older women with early breast cancer-a systematic review of literature. *World Journal of Surgical Oncology*.

[47] Aristei C., Kaidar-Person O., Tagliaferri L. (2018). The assisi think tank meeting and survey of post MAstectomy radiation therapy after breast reconstruction: the ATTM-SMART report. *European Journal of Surgical Oncology*.

[48] Montero-Luis A., Aristei C., Meattini I. (2019). The assisi think tank Meeting survey of post-mastectomy radiation therapy in ductal carcinoma in situ: suggestions for routine practice. *Critical Reviews in Oncology/Hematology*.

[49] Early Breast Cancer Trialists’ Collaborative Group (EBCTCG) (2011). Relevance of breast cancer hormone receptors and other factors to the efficacy of adjuvant tamoxifen: patient-level meta-analysis of randomised trials. *The Lancet*.

[50] Rodrigues N. A., Dillon D., Carter D., Parisot N., Haffty B. G. (2003). Differences in the pathologic and molecular features of intraductal breast carcinoma between younger and older women. *Cancer*.

[51] Gennari R., Curigliano G., Rotmensz N. (2004). Breast carcinoma in elderly women. *Cancer*.

[52] Diab S. G., Elledge R. M., Clark G. M. (2001). Tumor characteristics and clinical outcome of elderly women with breast cancer. *Journal of the National Cancer Institute*.

[53] Winzer K.-J., Sauerbrei W., Braun M. (2010). Radiation therapy and tamoxifen after breast-conserving surgery: updated results of a 2×2 randomised clinical trial in patients with low risk of recurrence. *European Journal of Cancer*.

[54] Shaitelman S. F., Khan A. J., Woodward W. A. (2014). Shortened radiation therapy schedules for early-stage breast cancer: a review of hypofractionated whole-breast irradiation and accelerated partial breast irradiation. *The Breast Journal*.

[55] Zhao H., Hei N., Wu Y. (2017). Initiation of and adherence to tamoxifen and aromatase inhibitor therapy among elderly women with ductal carcinoma in situ. *Cancer*.

[56] Chlebowski R. T., Kim J., Haque R. (2014). Adherence to endocrine therapy in breast cancer adjuvant and prevention settings. *Cancer Prevention Research*.

[57] Mukesh M., Harris E., Jena R., Evans P., Coles C. (2012). Relationship between irradiated breast volume and late normal tissue complications: a systematic review. *Radiotherapy and Oncology*.

[58] Tagliaferri L., Lancellotta V., Zinicola T. (2019). Cosmetic assessment in brachytherapy (interventional radiotherapy) for breast cancer: a multidisciplinary review. *Brachytherapy*.

[59] Albuquerque K., Tell D., Lobo P., Millbrandt L., Mathews H. L., Janusek L. W. (2012). Impact of partial versus whole breast radiation therapy on fatigue, perceived stress, quality of life and natural killer cell activity in women with breast cancer. *BMC Cancer*.

[60] Kawase E., Karasawa K., Shimotsu S. (2012). Estimation of anxiety and depression in patients with early stage breast cancer before and after radiation therapy. *Breast Cancer*.

[61] Farrow D. C., Hunt W. C., Samet J. M. (1992). Geographic variation in the treatment of localized breast cancer. *New England Journal of Medicine*.

[62] Autorino R., Vicenzi L., Tagliaferri V., Soatti C., Kovacs P. G., Aristei C. (2018). A national survey of AIRO (Italian Association of Radiation Oncology) brachytherapy (Interventional Radiotherapy) study group. *Journal of Contemporary Brachytherapy*.

[63] Tagliaferri L., Kovács G., Aristei C. (2018). Current state of interventional radiotherapy (brachytherapy) education in Italy: results of the INTERACTS survey. *Journal of Contemporary Brachytherapy*.

[64] Suh W. W., Pierce L. J., Vicini F. A., Hayman J. A. (2005). A cost comparison analysis of partial versus whole-breast irradiation after breast-conserving surgery for early-stage breast cancer. *International Journal of Radiation Oncology∗Biology∗Physics*.

[65] Tagliaferri L., Gobitti C., Colloca G. F. (2018). A new standardized data collection system for interdisciplinary thyroid cancer management: thyroid COBRA. *European Journal of Internal Medicine*.

[66] Tagliaferri L., Kovács G., Autorino R. (2016). ENT COBRA (Consortium for Brachytherapy Data Analysis): interdisciplinary standardized data collection system for head and neck patients treated with interventional radiotherapy (brachytherapy). *Journal of Contemporary Brachytherapy*.

[67] Tagliaferri L., Budrukkar A., Lenkowicz J. (2018). Ent cobra ontology: the covariates classification system proposed by the Head & Neck and Skin GEC-ESTRO Working Group for interdisciplinary standardized data collection in head and neck patient cohorts treated with interventional radiotherapy (brachytherapy). *Journal of Contemporary Brachytherapy*.

[68] Tagliaferri L., Pagliara M. M., Boldrini L. (2017). INTERACTS (INTErventional Radiotherapy ACtive Teaching School) guidelines for quality assurance in choroidal melanoma interventional radiotherapy (brachytherapy) procedures. *Journal of Contemporary Brachytherapy*.

[69] Meldolesi E., van Soest J., Damiani A. (2016). Standardized data collection to build prediction models in oncology: a prototype for rectal cancer. *Future Oncology*.

